# A Novel Stay-Green Mutant of Rice with Delayed Leaf Senescence and Better Harvest Index Confers Drought Tolerance

**DOI:** 10.3390/plants8100375

**Published:** 2019-09-26

**Authors:** M. K. Ramkumar, S. Senthil Kumar, Kishor Gaikwad, Rakesh Pandey, Viswanathan Chinnusamy, Nagendra Kumar Singh, Ashok Kumar Singh, Trilochan Mohapatra, Amitha Mithra Sevanthi

**Affiliations:** 1 ICAR-National Institute for Plant Biotechnology, New Delhi 110012, India; ramcena619@gmail.com (M.K.R.); kish2012@gmail.com (K.G.); nksingh4@gmail.com (N.K.S.); 2 PG and Research Department of Botany, National College, Tiruchirapalli 620001, India; senthil@nct.ac.in; 3 ICAR-Indian Agricultural Research Institute, New Delhi 110012, India; r_pan_pdcsr@yahoo.co.in (R.P.); viswa.chinnusamy@gmail.com (V.C.); aks_gene@yahoo.com (A.K.S.); 4 Indian Council of Agricultural Research, Krishi Bhawan, New Delhi 110001, India; dg.icar@nic.in

**Keywords:** drought tolerance, EMS induced mutants, Nagina 22, rice, stay-green trait

## Abstract

Three Ethyl methansulphonate (EMS)-induced stay-green mutants (SGM-1, SGM-2 and SGM-3) and their wild-type (WT), were tested for their Stay-Green (SG) and drought tolerance nature as the relation between these two attributes is not yet established in rice. In the dark induced senescence assay, SGM-3 showed delayed senescence while SGM-1 and SGM-2 showed complete lack of senescence. Mutants showed stable transcript abundance over time, for 15 candidate genes (CGs) associated with senescence, compared to the WT. SGM-3 however showed moderately increasing transcript abundance over time for *ATG6a*, *ATG4a*, *NYC1*, *NOL* and *NYC3*. Only SGM-3 performed better than the WT for yield and harvest index under well irrigated as well as drought conditions, though all the mutants showed better performance for other agronomic traits under both the conditions and ascorbate peroxidase activity under drought. Thus, SG trait showed positive correlation with drought tolerance though only SGM-3 could convert this into higher harvest index. Sequence analysis of 80 senescence-associated genes including the 15 CGs showed non-synonymous mutations in four and six genes in SGM-1 and SGM-2 respectively, while no SNPs were found in SGM-3. Analysis of the earlier reported Quantitative Trait Loci (QTL) regions in SGM-3 revealed negligible variations from WT, suggesting it to be a novel SG mutant.

## 1. Introduction

Constant decline in the availability of water resources on one hand and increased utilization of the same resources on the other hand has made drought stress a real threat to global food security [1,2]. Plants, being rooted to the ground, have developed tolerance mechanisms to drought stress by altering their morphological, physiological and molecular mechanisms [3,4,5]. The magnitude of drought stress experienced by a plant depends on the growth stage of the plant and the duration of the stress. Rice (*Oryza sativa* L.), a monocot with shallow root architecture but huge water requirement for cultivation—is more prone to water deficit stress than other major food crops [6]. Though rice is vulnerable to drought stress, invariably, across all the growth stages, reproductive stage stress causes more adverse effects as grain yield gets compromised [7]. Drought stress destabilizes the photosynthetic machinery, leading to fluctuations in the carbon and nitrogen metabolism ultimately resulting in reduced sink size [8]. Drought tolerance is attained by two phenomena: by completing the life cycle in a shorter duration (escape) or by developing morpho-physiological adaptations such as root architecture, water use efficiency, osmoregulation, stomatal and non-stomatal limitations and molecular changes to withstand the stress condition (true tolerance) [9,10,11,12].

Since chlorophyll, the most abundant pigment on earth, is a phototoxic compound, it is degraded by a highly conserved autophagy mechanism in plants [13,14]. Plant autophagy plays a crucial role in the onset of leaf senescence while certain autophagy genes are also involved in stress response [15,16,17]. Leaf senescence, the final stage of plant development, wherein the shift from the carbon capture phase to the nitrogen remobilization phase occurs as the final source of nutrient supply to the developing grains, is a highly regulated molecular mechanism [18,19]. Delayed senescence, i.e., stay-green (SG) phenotype, associated with elevated levels of cytokinins showed drought tolerance capacity without compromising yield [20,21]. SG trait can also be a result of better maintenance of water intake and supply equilibrium after anthesis [22]. Adverse impact of drought stress can be partially mitigated by enhanced photosynthetic activity during post-anthesis [23]. Thus mutants with delayed senescence that exhibit prolonged photosynthetic activity can have enhanced productivity and better drought tolerance as a result of better water maintenance. SG mutants with retention of photosynthetic activity are termed as functional while those without the photosynthetic activity are termed as cosmetic mutants [24].

SG phenomenon have been studied in a wide range of crops such as sorghum, maize, wheat, barley, soybean, potato, rice etc., [21,25,26,27,28,29,30,31]. In cereals such as sorghum, maize and wheat, functional SG traits have been reported [32]. Four major QTLs, i.e., Stg1–4, responsible for stay-greenness were reported to retain larger green leaf area under drought environment during grain filling in sorghum [33,34]. Positive correlation between delayed leaf senescence and higher grain yield and higher post-anthesis biomass under drought stress was also reported in Sorghum [35,36]. In wheat, the functional SG mutants showed enhanced antioxidants defense mechanism and significant grain yield under drought stress [29,37,38,39]. In rice, so far, such a relationship between drought tolerance and SG trait has not been explored. Even under well irrigated conditions, functional stay-green mutants are of greater value as they possess yield enhancing qualities. Several QTLs, e.g., rdgf2a, rdgf2b, rdgf3, rdgf8a, rdgf9, rdgf10, qCCAI-9, qCCAJ-9, qRCRJ-9, etc., responsible for SG phenotype have been mapped in rice, but they have been reported for their negative correlation with yield [40,41,42]. Further, a recessive stay-green allele, *sgr* has been mapped on chromosome 9 which showed reduced photosynthetic activity [43,44]. Another SG mutant reported showed decrease in the chlorophyll a and other chlorophyll protein complexes but retention of chlorophyll b and LHC (light harvesting complex) II [45]. Both these mutants had impairment in chlorophyll catabolism and hence were cosmetic SG mutants. A functional stay-green mutant, SNU-SG1 was derived from a japonica cultivar by EMS induced mutagenesis. This mutant showed delayed decline in the rate of photosynthesis and chlorophyll content compared to some of the elite japonica cultivars. Though QTLs for the functional stay-greenness in this mutant have been mapped on chromosome 9, neither the molecular basis nor its drought tolerance ability is yet known [46]. The present study was undertaken with the objective of characterizing three EMS induced mutants for their stay-greenness, and to know whether the SG trait improved the agronomic performance and led to enhanced drought tolerance in rice as in the case of other cereals like sorghum, wheat and maize.

## 2. Results

### 2.1. Characterization of the SG Mutants

Three of the six stay-green mutants (SGM-1, SGM-2 and SGM-3), which showed 90% or more similarity for DUS (Distinctness, Uniformity and Stability) index and monomorphic for 72 genome-wide Simple Sequence Repeat (SSR) markers as compared to their WT, Nagina 22 (N22) (Table S1), were selected for further studies. These results validated the trueness of the mutant phenotype to their WT.

### 2.2. Dark Induced Senescence (DIS) Assay of the SG Mutants

**Figure 1:**
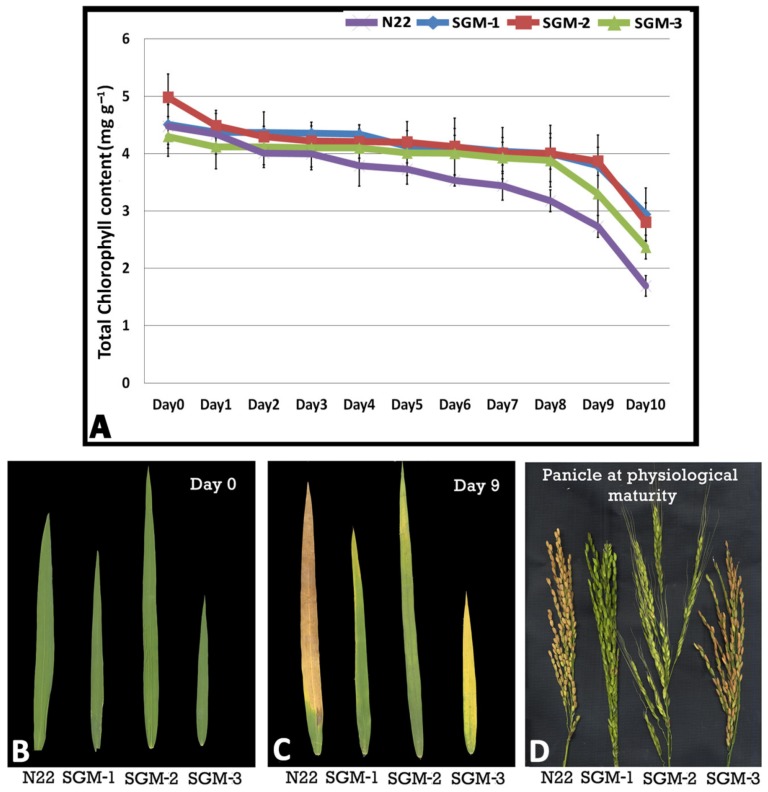
Senescence in the three stay-green mutants (SGM-1 to SGM-3) and the wild type, Nagina 22 (N22). (**A**) Total chlorophyll content in the flag leaf of the mutants and N22 under dark induced senescence from day 0 to day 10; (**B**) flag leaf images of the mutants and N22 on day 0; (**C**) flag leaf images of the mutants and N22 on day 10; (**D**) panicles of the mutants and N22 at physiological maturity.

The initial chlorophyll content (day 0) was the highest in the mutant, SGM-2 (4.97 mg·g^−1^) followed by SGM-1 and N22 with nearly equal chlorophyll content (~4.5 mg·g^−1^) while SGM-3 had the least chlorophyll content (Figure 1A). The rate of decline in chlorophyll content was gradual and the slowest in SGM-3 which showed signs of deep decline only on day 8. SGM-2 showed a sharp decline in chlorophyll content on day 2 and on day 8. SGM-3 was nearly similar to N22 in appearance on day 10 while the other two mutants remained green even on day 10 (Figure 1B,C). Invariably, all the three mutants had higher chlorophyll content than the wild type (WT) on day 10. Thus, all the three mutants proved to be of stay-green type by the DIS assay (Figure 1A). Further, on physiological maturity, SGM-1 and SGM-2 panicles remained completely green while SGM-3 turned brown with some tinges of greenness (Figure 1D). While SGM-1 and SGM-2 showed complete lack of senescence, suggesting impairment in chlorophyll metabolism, SGM-3 showed delayed senescence (Figure 1B–D).

### 2.3. Measurement of Photosynthetic Activity

**Figure 2:**
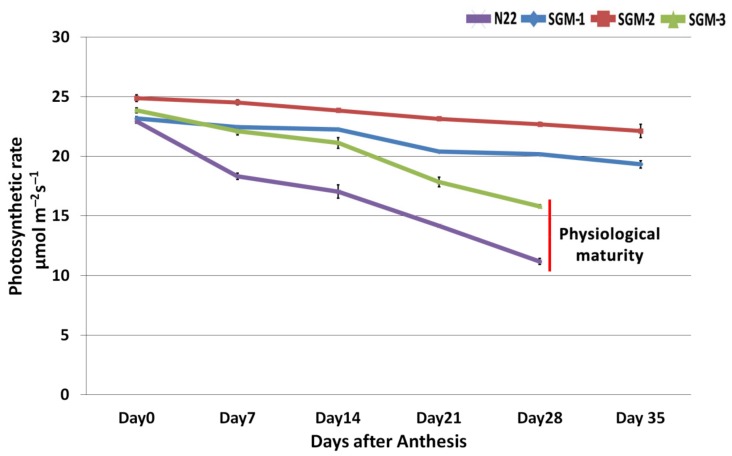
Photosynthetic rate of the mutants and the wild type Nagina 22 (N22) post-anthesis till physiological maturity. While N22 and SGM-3 reached physiological maturity in 28 days post-anthesis SGM-1 and SGM-2 took 35 days.

While N22 and SGM-3 were similar in days to heading and physiological maturity, the other two mutants were late by 10 days. Hence the mutants SGM-2 and SGM-3 had six data points (day 1 to day 35 at weekly intervals), while N22 and SGM-1 had only five data points (till day 28). The photosynthetic rate of the SG mutants and the WT was almost similar, ranging from 23–25 µmol m^−2^ s^−1^ at the time of anthesis (Figure 2). A steep and steady decline in the photosynthetic activity was observed in WT post-anthesis which dropped to as low as 11 µmol m^−2^ s^−1^ at the time of physiological maturity. However, the mutants, except SGM-3, showed negligible levels of reduction and retained their photosynthetic activity on the whole, showing a decline of 2–3 µmol m^−2^ s^−1^ at maturity. In SGM-3, the photosynthetic activity remained steady till day 14 but then gradually reduced and reached 15 µmol m^−2^ s^−1^ by 28 days. Considering the photosynthetic activity and senescence data together, SGM-1 and SGM-2 were active in their photosynthesis but did not show any senescence, while SGM-3 showed gradual reduction in photosynthetic activity and underwent delayed senescence.

### 2.4. Time-Course Expression Analysis of Chlorophyll Catabolism and Senescence Related Genes in the SG Mutants under Well-Watered Conditions

**Figure 3:**
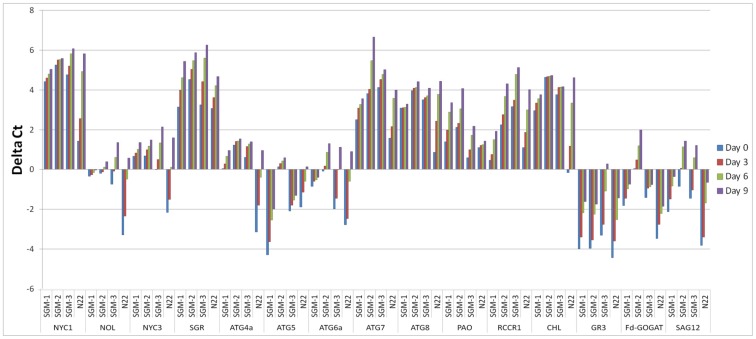
Time-course relative gene expression analysis of 15 genes involved in chlorophyll catabolism, senescence and nitrogen assimilation in three stay-green mutants (SGM-1 to SGM-3) and the wild type, Nagina 22 (N22) under dark incubation.

Time-course analysis of relative gene expression was undertaken under well-watered DIS conditions for 15 genes known to be involved in chlorophyll catabolism and senescence. Seven of the 15 genes, namely, Non Yellow Coloring 1 Like (*NOL*), Stay Green (*SGR*), Pheophorbide a Oxygenase (*PAO*), Red chlorophyll catabolite reductase 1 (*RCCR1*) and three autophagy related genes Autophagy 6a(*ATG6a*), Autophagy 7 (*ATG7*) and Autophagy 8 (*ATG8*), showed upregulation while five genes, namely, *NOL*, Senescence Associated Gene 12 (*SAG12*), Glutathione reductase 3 (*GR3*), Ferredoxin-dependent glutamate synthase (*Fd-GOGAT*) and Autophagy 5 (*ATG5*) showed downregulation in all the four lines for most of the points in the time-course (Figure 3). Exceptions for this include Autophagy 4a (*ATG4a*) and Chlorophyllase (*CHL*) genes which showed upregulation in all the three SG mutants, but downregulation in the WT. Further, for all the 15 genes studied, the mutants as well as the WT showed an increasing trend in gene expression with the progression of time under incubation, irrespective of whether they are up or downregulated, i.e., the expression of the genes increased as the time under dark condition increased and this rate of increase was the highest in the WT followed by SGM-3, while SGM-1 and SGM-2 were at par showing the least rate of increase (Figure 3 and Table S2). Another important observation was that in the case of upregulated genes, the mutants showed higher level of upregulation from the beginning itself while the WT showed gradual upregulation (Figure 3). Similarly, in the case of downregulated genes, in the WT, the degree of downregulation was higher in the beginning which gradually matched the mutant levels on day 9. Thus, the SGMs had a considerably stable level of expression at different time points compared to the WT. Among the SG mutants, SGM-3 was different by showing a moderately increasing trend of transcript level over the time-course, partially mimicking the WT, especially in the case of *ATG6a*, *ATG4a*, *NYC1*, *NOL* and *NYC3*. Further, by the end of the time-course, the gene expression levels were nearly uniform for many genes among the mutants as well as N22. For instance, in the case of *NYC1*, *NOL* and *NYC3* uniform transcript levels were reached on day 6 in the mutants and the WT while in the case of *ATG4a* uniform levels were attained on day 9. SGM-3 remained an exception by showing elevated expression levels in a few autophagy related genes and the genes involved in chlorophyll catabolism, similar to the WT. Overall, the expression profiles suggested that chlorophyll catabolism and senescence were severely impaired or delayed in SGM-1 and SGM-2 but moderately so in SGM-3, compared to the WT.

### 2.5. Physiological and Agronomic Performance of the Stay-Green Mutants under Drought Stress Compared to the Well-Irrigated Conditions

**Figure 4:**
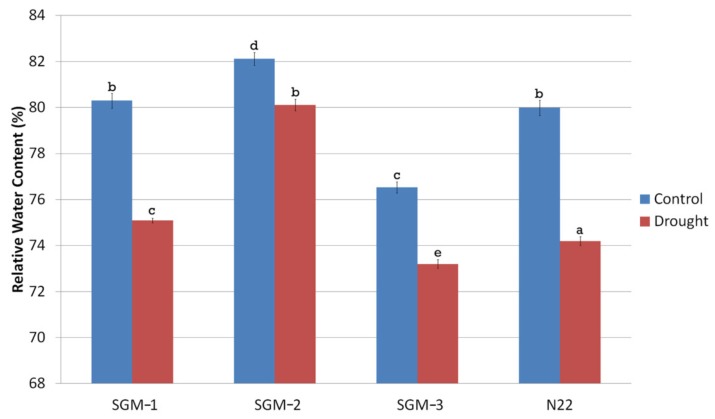
Relative water content (%) of the three stay-green mutants (SGM-1 to SGM-3) and the wild type, Nagina 22 (N22) under well-watered (control) and drought stress conditions (a to e denotes significance at *p* (0.05) determined from one way ANOVA post hoc Tukey test).

**Figure 5:**
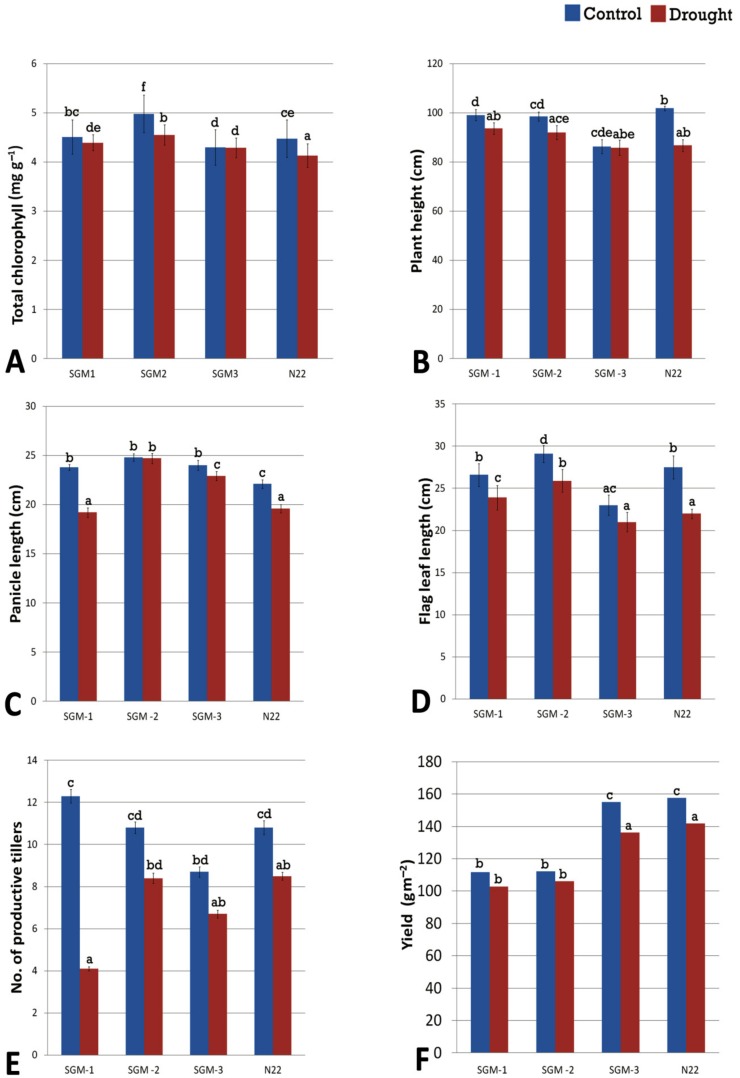
Performance of the three stay-green mutants (SGM-1 to SGM-3) and the wild type, Nagina 22 (N22) for (**A**) chlorophyll content, (**B**) plant height, (**C**) panicle length, (**D**) flag leaf length, (**E**) number of productive tillers, (**F**) plot yield under well-watered (control) and drought stress conditions (a to e denotes significance at *p* (0.05) determined from one way ANOVA post hoc Tukey test).

The gravimetric soil moisture content of the experiment field under drought stress ranged from 11.5–12% while that of the well-watered field was 30%. Under drought, the Relative Water Content (RWC) of all the four lines was significantly different from that of well-irrigated control (Figure 4). SGM-2 had the highest RWC under both the treatments (82.2% under well-irrigated and 80% under drought stress) and it also showed the lowest reduction in RWC (2.5%) under drought stress. SGM-3 though had the lowest RWC under both the treatments the % reduction in RWC under stress was minimal (4%). Though N22 and SGM-1 had similar RWC under well-irrigated (~80%) and drought stress (75%) conditions, they showed steep decline in RWC under stress (7.5% and 6.5% respectively). SGM-3 retained its chlorophyll content almost unchanged under drought stress, while the other two mutants showed minimal differences. Overall, under drought, the change in chlorophyll content was not significant in the mutants while WT showed significant decline (Figure 5A). For the agronomic traits also, the WT showed a significant reduction in performance under drought treatment. However, the SG mutants behaved differently for different traits (Figure 5B–F). For instance, drought did not have any influence on plant height of the SG mutants (Figure 5B). Moreover, SGM-2 and SGM-3 did not show any alterations in number of productive tillers (NPT) under drought. However, the most important trait, yield/plot (g m^−2^), indicated that only the WT and SGM-3 performed better under drought stress though there was a decline in their performance (~130 g m^−2^) compared to the irrigated control (~150 g m^−2^). The other two SG mutants, SGM-1 and SGM-2 showed comparatively poorer performance under both the treatments for yield (100–110 g m^−2^), though they did not show any reduction in performance under drought stress compared to the control conditions. Moreover, under well irrigated condition, the harvest index (HI), of SGM-3 was the highest (56%) compared to the WT, N22 (45%) and the other two SG mutants (31 and 33%). This observation clearly established the agronomic superiority of SGM-3 over the other two mutants which showed delayed senescence, better photosynthetic activity post-anthesis and no impairment in source–sink translocation of photosynthetic resources. Thus, the three SG mutants performed better than the WT under drought stress in terms of both the physiological and agronomic parameters but SGM-1 and SGM-2 could not translate it into yield while SGM-3 showed the best performance on that front too with the highest harvest index.

### 2.6. Oxidative Stress Associated Enzyme Studies on the Mutants under Drought Stress

**Figure 6:**
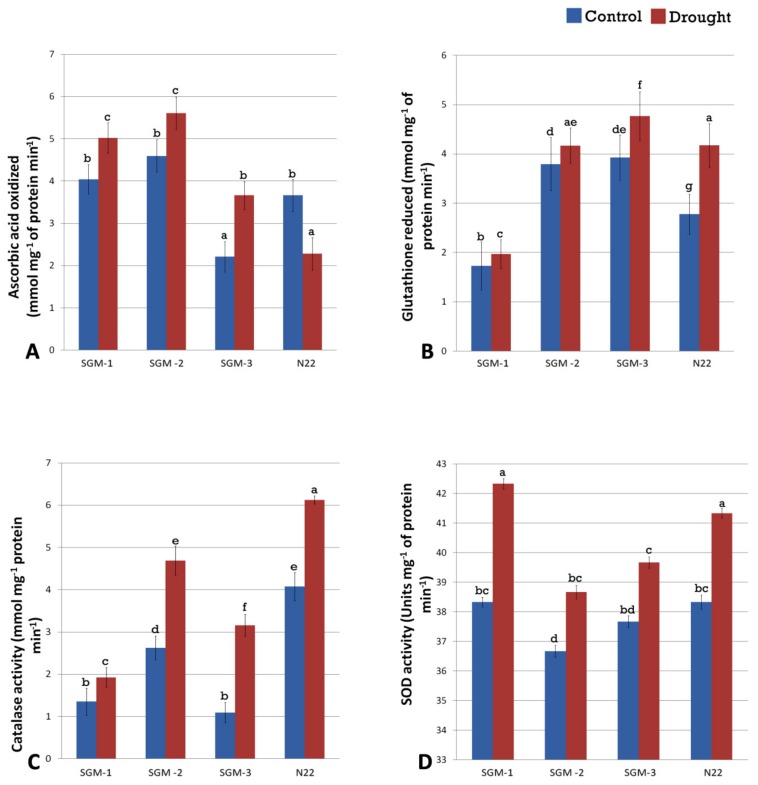
Enzyme activities of the enzymes involved in oxidative stress management, (**A**) ascorbate peroxidase (APx), (**B**) glutathione reductase (GR), (**C**) catalase (CAT) and (**D**) superoxide dismutase (SOD) in the three stay-green mutants (SGM-1 to SGM-3) and the wild type, Nagina 22 (N22) (a to f denotes significance at *p* (0.05) determined from one way ANOVA post hoc Tukey test).

All the four oxidative stress management (OSM) related enzymes, Ascorbate peroxidase (APx), Glutathione reductase (GR), Superoxide Dismutase (SOD), and Catalase (CAT) showed enhanced activity under drought stress compared to well-watered control conditions in the three mutants and the WT showing that all the four lines had better OSM under drought (Figure 6A–D). The only exception was the WT for APx activity. In terms of enzyme activity under drought stress, no definite hierarchy in performance emerged among the mutants and the WT could be found; as for different enzymes, different lines performed the best. For APx enzyme, SGM-1 and SGM-2 showed the highest activity while for GR the highest activity was seen in SGM-3. N22, a drought tolerant genotype, performed better than the mutants with respect to catalase activity whereas for SOD, SGM-1 and N22 activity were on par (Figure 6C,D). Comparison in terms of ‘fold increase in enzyme activity under drought’ clearly showed that SGM-3 was the best performer, as it had the highest fold increase for APx (1.65) and GR (2.89) and the second best fold increase for SOD activity, compared to the enzyme activity under irrigated conditions (Table S3).

### 2.7. Expression Analysis of the Chlorophyll Catabolism and Senescence Related Genes under Drought

**Figure 7:**
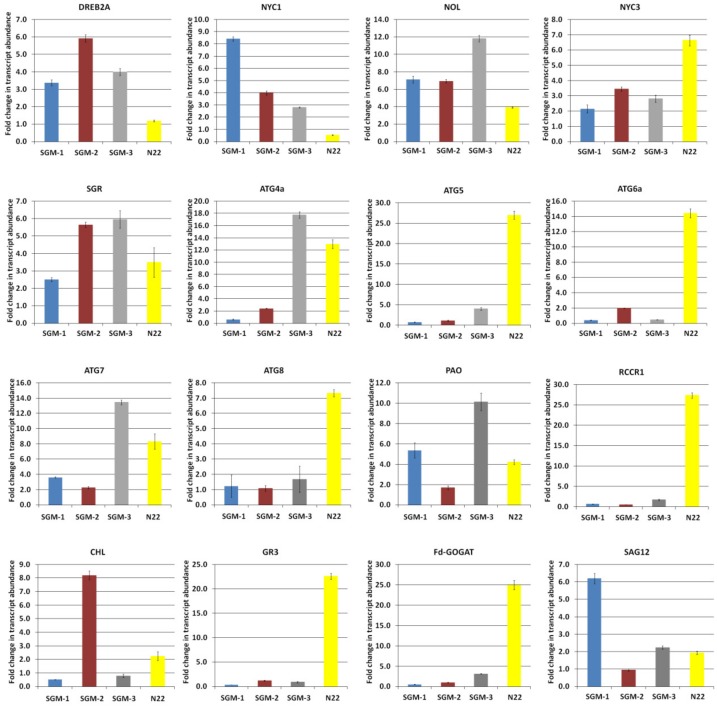
Relative gene expression analysis of *DREB2A* and 15 genes involved in chlorophyll catabolism, senescence and nitrogen assimilation in three stay-green mutants (SGM-1 to SGM-3) and the wild type, Nagina 22 (N22) under drought stress.

Upregulation of dehydration-responsive element binding protein 2(*DREB2A*) in all the four genotypes under drought condition indicated that drought stress was really experienced by the plants (Figure 7). Further, all the 15 genes showed upregulation from less than one-fold (0.3) to 27.4-fold across the SG mutants and the WT, indicating moderate to high response to drought (Figure 7). Among the five autophagy associated genes, N22 showed drastic upregulation in all the five genes (8- to 27-fold) compared to the mutants, except for SGM-3 in the case of *ATG4a* and *ATG7*. In both these genes, SGM-3 showed more upregulation (13-fold) than N22 (7–8-fold). SGM-3 also had the highest upregulation for *NOL*, *SGR* and *PAO* while SGM-1 showed the highest expression for *NYC1* and *SAG12*. SGM-2 showed the highest upregulation only for *CHL*. Further, GR involved in OSM and Fd-GOGAT involved in nitrogen remobilization showed higher expression in N22 than in the mutants. Thus, overall SGM-3 and N22 differed from the other two mutants in their expression of genes associated with senescence and chlorophyll catabolism under drought.

### 2.8. Sequence Analysis

To better understand the delayed senescence and chlorophyll retention nature of the mutants, the sequence analysis of the 15 candidate genes was carried out with respect to N22. SGM-1 had 40 SNPs dispersed across four genes (*NYC1*, *NYC3*, *ATG5* and *Fd-GOGAT*) out of which 8 resulted in non-synonymous amino acid changes (Table 1 and Supplementary Table S4). The maximum number of these amino acid changes (5/8) was present in *Fd-GOGAT*. In SGM-2, a total of 54 SNPs were identified across 6 genes of which *Fd-GOGAT* and *NYC3* were common with SGM-1 with identical SNPs. Apart from these two genes, SGM-2 had mutations in *SGR*, *RCCR1*, *CHL*, and *GR* with the total amino acid changes numbering to 22. *Fd-GOGAT* and *CHL* had the highest number of amino acid substitutions followed by *RCCR1* and *SGR*. Interestingly, *RCCR1* had the stop codon at position 330 (SNP position 990) in the place of phenylalanine, leading to truncated protein in SGM-2 (Table S4). This mutant had more number of mutated genes, mostly those involved in the chlorophyll catabolism. More interestingly, SGM-3 had no SNPs in these 15 genes with sequences identical to that of N22, thus validating the results obtained in the expression analysis. Out of the 65 additional genes analyzed for sequence variations, five genes had non synonymous mutations, i.e.: four genes in SGM-1, two in SGM-2 and one in SGM-3 (Table S5). In SGM-2, a splice variant of *NAC5* had extended protein due to the mutation at the stop codon position 901. Multiple mutations were observed in *GSTF10* of SGM-1. All three mutants including SGM-3 had a single non-synonymous mutation in *ACC oxidase*.

## 3. Discussion

All the three mutants showed enhanced chlorophyll retention under dark incubation compared to the WT. While SGM-1 and SGM-2 retained higher levels of chlorophyll throughout the DIS time period, SGM-3 maintained a consistent level followed by a decline at the end. The mutants had photosynthetic rate equivalent to N22 at anthesis. The decline in photosynthetic rate post-anthesis till physiological maturity was insignificant in SGM-1 and SGM-2, whereas SGM-3 exhibited delayed leaf senescence. This phenomenon elucidates the nature of the cosmetic and functional stay-green phenotype respectively [47]. Though the SGM-3 mutant was smaller in stature than N22, with significant reduction in PH, FLL and NPT, it did have grain yield equivalent to the WT, N22 under irrigated conditions (Figure 4) thereby leading to a higher harvest index than the WT (56% against 45%). On the other hand, SGM-1 and SGM-2 with bigger stature than the WT, produced grain yield much lesser than the WT. Such an increase in biomass without accompanied increase in grain yield has been reported in rice transgenics with delayed leaf senescence [48] and this could be a typical feature of cosmetic SG mutants [24]. Thus, SGM-1 and SGM-2 were inferred as cosmetic SG mutants while SGM-3 as a functional SG mutant.

For biomass and physiological traits, the SG mutants showed least reduction in their performance under drought stress compared to control. Hence, the SG mutants were certainly better than the WT for the secondary traits under drought stress. As SGM-3 alone is a functional SG mutant, it could perform equally well for grain yield too under drought. Among the wide range of mechanisms contributing towards drought tolerance and leaf senescence, drastic increase in the ROS levels due to alterations in the electron transport chain leading to cell death is one of them [49,50]. Nagina 22 is known for its better oxidative stress management mechanism under drought [13,39,51]. It was demonstrated by Prakash et al. (2016) [51] that N22 had better response for SOD and GR but not for APx through enzyme activity, gene sequence and protein stability studies. In the current study, the same trend was found with additional information on catalase in which again N22 was found to give better response under drought. Though the SG mutants more or less had the same response akin to N22 under drought for these enzymes, they did show drastic enhancement in APx activity suggesting that rice SG mutants do have better drought tolerance, as found in sorghum and wheat [52]. SGM-3 mutant had the best expression for GR under dark incubation in the time-course analysis through expression and enzyme activity studies, compared to the other SG mutants and the WT, indicating that the oxidative damage during senescence was very limited in this mutant.

*Fd-GOGAT* is the primary enzyme for photorespiration and N assimilation in leaves [53] for which SGM-2 performed the poorest under dark incubation while both SGM-1 and SGM-2 showed poor performance under drought. Only SGM-3 showed a moderate performance for this enzyme, showing that the N assimilation in this mutant is functional. The contrasting transcript level for the autophagy related genes in SGM-1 and SGM-2, compared to N22 clearly showed that senescence is severely impaired in these mutants. As leaf senescence is a major determinant of yield [22], the impairment in senescence resulted in their low yield potential. In contrast, the transcript levels of SGM-3 matched that of N22 for the autophagy related genes, especially, in the latter part of time-course analysis showing that senescence is delayed in this mutant but not impaired which was also corroborated by its better performance in grain yield.

Sequence analyses provided strength to our results showing that the chlorophyll catabolism was impaired in SGM-2, which produced a non-functional protein product of *RCCR1*. This gene is known to catalyze the conversion of pheophorbide a to pFCC, i.e., primary flourescent chlorophyll catabolite, in the catabolism of chlorophyll a [47]. As chlorophyll disintegration was one of the first visible symptoms of drought stress, the drought stress expression profiling of SGM-2 was very interesting with the lowest transcript abundance for *RCCR1* but with highest abundance of *CHL* (chlorophyllase) which catalyzes the first step of chlorophyll a catabolism (chlorophyll a to chlorophyllide a). Further, both SGM-1 and SGM-2 had a lot of non-synonymous amino acid changes in *Fd-GOGAT*, giving strength to their impaired N assimilation and lower grain yield even under irrigated conditions. These two mutants also had common mutations in *NYC-3* gene with identical expression profiles in the DIS time-course analysis showing their non-functional SG nature wherein the chlorophyll catabolism is compromised. On the other hand, SGM-3 did not have any sequence variations for any of these 15 genes. We also found multiple non-synonymous mutations in SGM-1 and SGM-2 for the 65 additional candidate genes tested. Since SGM-3 behaved like a functional mutant in our study with better performance under drought and showed no variations in the 80 candidate genes tested other than a single non-synonymous mutation in *ACC oxidase*, which was again common to all the 3 mutants, we looked for mutations within the only known main effect QTL on chromosome 9 reported from the functional SG mutant, SNU-SG1 [46]. Out of the 428 genes present in the QTL region, 31 had non synonymous SNPs (Table S6). Protein stability studies (in silico) of these 31 gene products did not show significant changes from the WT suggesting that SGM-3 could be a novel and functional SG mutant. Further efforts towards mapping the causal gene in SGM-3 would not only contribute towards understanding of the SG trait but also would help in rice improvement as the prolonged photosynthesis and enhanced drought tolerance in this mutant can lead to better yield both under well-irrigated and drought stress conditions, especially in genotypes with larger source and sink sizes. Hence, in our laboratory, we are in the process of mapping this mutant and breeding it by crossing it with genotypes with high biomass and sink size.

## 4. Materials and Methods

### 4.1. Plant Materials and Dark Induced Senescence Assay

From the national repository of EMS mutant resources of Nagina 22 (N22), specifically from the mutant garden maintained at ICAR-National Institute for Plant Biotechnology (NIPB), New Delhi, six stay-green (SG) mutants in M_7_ generation were selected based on their morphological appearances, i.e., dark green leaves and delayed senescence in the field [29,54] (http://14.139.229.201/EMSgardeN22/). Since EMS-induced mutants harbor a huge number of background mutations, we tested the genetic background of these mutants using 72 genome-wide SSR markers [5] followed by morphological characterization following the national guidelines for the conduct of tests for distinctness, uniformity and stability (DUS) [55]. To ascertain the SG nature of the three mutants selected based on SSR and DUS similarity, namely, SGM-1, SGM-2 and SGM-3, dark induced senescence (DIS) assay was done post anthesis [18,56]. For all experiments, WT served as a control. For DIS assay, flag leaf samples were collected after anthesis in three biological replicates from the mutants and WT grown in 1 m^2^ blocks at the experimental farm of Indian Agricultural Research Institute (IARI), New Delhi. The flag leaf samples were incubated in dark condition at room temperature for 10 days. Chlorophyll content was measured at every 24 h intervals for 10 days (day 0–day 9). For estimation of chlorophyll a and b, 5 mg of leaf sample was taken and incubated in 1 mL of DMSO (Dimethyl Sulfoxide) for 24 h in dark. Absorbance was measured at 645 nm and 663 nm using a 96 well plate reader (Varioskan™, Thermo Scientific, Waltham, MA, USA). The chlorophyll content was calculated using Arnon’s equation [5,57].

### 4.2. Measurement of Photosynthetic Activity

To understand the functional stay-greenness of the mutants, four individual flag leaves were labeled at heading stage to measure the photosynthetic activity of SG mutants and WT. The net photosynthetic rate was measured using portable photosynthesis system (Li-6400XT, LI-COR Biosciences, Lincoln, NE, USA) at 10:30 am–12:30 pm, under 1200 μmol photon m^−2^·s^−1^ light intensity, post-anthesis till physiological maturity in seven days interval. During the entire time course of the observations, the atmospheric CO_2_ concentration, air temperature, and relative air humidity were 390–400 μmol mol^−1^, 35–38 °C, and 46–52%, respectively.

### 4.3. Sampling for Candidate Gene Expression Profiling under DIS

In order to study the expression pattern of the candidate genes under senescence, it was important to conduct the DIS assay without detaching the flag leaf from the plant. To provide dark environment to the flag leaves in vivo, a modified protocol of Weaver et al. (2001) was followed [58]. A scaffold was made using aluminum foil around the flag leaf which was wrapped by butter paper to minimize the high temperature effect on the leaf. Sampling was done on day 0, day 3, day 6 and day 9 in three biological replicates.

### 4.4. Drought Stress Treatment and Sampling

To examine the drought tolerance capacity of the three SG mutants they were grown along with the WT in 1 m^2^ blocks at the rain-out shelter facility of NIPB, IARI, New Delhi during Kharif (June–September) 2016. Drought stress was imposed at booting stage by withholding water for 10 days. The gravimetric soil moisture content was measured by collecting the soil sample from a depth of 60 cm at multiple sites of the experiment field [59]. Flag leaf samples were collected after the drought stress period for gene expression analysis and enzymatic studies. For comparison of gene expression and enzyme activity under drought, flag leaf samples from the well-irrigated control plants grown in the IARI farm, but not subjected to DIS assay were used.

### 4.5. Measurement of Agronomic, Physiological Traits

To study the performance of the mutants, agronomic characters such as plant height, panicle length, flag leaf length, number of productive tillers (NPT), yield per plot and harvest index (HI) were recorded. Under stress condition, along with these agronomic traits, the physiological parameters like Relative Water Content (RWC) and chlorophyll content were also noted. All agronomic traits except HI were recorded at physiological maturity. HI was calculated as ratio of grain weight to shoot biomass and expressed in percentage. HI is presented only for well-irrigated control. Since the shoot biomass samples from the drought stress plot were damaged before weighing, reliable estimates of HI under drought stress could not be obtained.

### 4.6. Enzyme Assay

Leaf extracts for superoxide dismutase (SOD; EC 1.15.1.1), glutathione reductase (GR; EC 1.6.4.2), ascorbate peroxidase (APX; EC 1.11.1.1) and catalase (EC 1.11.7.6) was prepared from the flag leaf of control and drought samples. The samples were pre-frozen in liquid nitrogen to prevent proteolytic activity, and ground in 3 mL extraction buffer containing 0.1 M phosphate buffer (pH 7.5) and 0.5 mM EDTA. Extracts were centrifuged at 15,000 *g* for 20 min at 4 °C and the supernatant was collected and used for the enzyme assay. The concentration of the extracted protein was estimated by Bradford method before performing enzyme assays. Each enzyme assay was scaled down to miniprep of 200 μL volume and the spectrophotometric measurements were done in a 96-well plate reader (Varioskan™, Thermo Scientific, Waltham, MA, USA). Enzyme assays were performed using three biological and three technical replicates. SOD, APX and GR activity were assayed and the activity of respective enzymes was calculated as explained in Prakash et al. (2016) [51]. Catalase (CAT) was assayed by measuring the disintegration of H_2_O_2_. The reaction mixture (200 μL) consisted of 10 μL of dilute enzyme extract and 100 μL of 0.1 M phosphate buffer (pH 7) and 60 μL of water. The reaction was initiated by adding 30 μL of 75 mM H_2_O_2_. A decrease in absorbance at 240 nm was observed every 30 s for 3 min with UV–visible spectrophotometer. The molar extinction coefficient of 39.4 M^−1^ cm^−1^ was used for the calculation of enzyme activity.

### 4.7. RNA Extraction and Gene Expression Profiling

For the expression analysis of senescence associated genes, 15 candidate genes were selected which included genes involved in chlorophyll catabolism (*NYC1*, *NYC3*, *NOL*, *SGR*, *PAO*, *RCCR1*, *CHL*), autophagy and senescence (*ATG4a*, *ATG5*, *ATG6a*, *ATG7*, *ATG8*, *Fd-GOGAT*, *SAG12*), genes associated with drought stress (*DREB2A*) and oxidative stress management (*GR*). The details of the primers used are given in Table S7.

Total RNA was isolated by Trizol method from flag leaves which were collected from drought treatment plots (sampled at 10 days after withholding water) and well-watered plots subjected to DIS assay on day 0, day 3, day 6 and day 9. DNase treatment was carried out using Turbo DNA DNase free (Ambion, Austin, TX, USA) according to manufacturer’s instruction. Integrity and quality of the RNA were checked on 1.5% agarose gel. RNA concentration was determined using Nanodrop, ND-8000 spectrophotometer (Thermo Fisher Scientific, Waltham, MA, USA). RNA samples with good quality and integrity were used for cDNA synthesis. For single stranded cDNA synthesis 1 μg of total RNA along with dNTP, reverse transcriptase (RT) enzyme, buffer, oligo-dT and hexamer primers from Superscript III first strand synthesis kit (Thermo Fisher Scientific, Waltham, MA, USA) were used. Semi-quantitative and quantitative RT-PCR was performed to analyze the expression profile of candidate genes. cDNA were diluted 5-fold and used as template for Semi-quantitative and qRT-PCR. Actin was used as the housekeeping gene for normalization. For semi-quantitative PCR, the following reaction condition was used: initial denaturation at 94 °C for 5 min, 30 cycle of 94 °C for 30 s, 60 °C for 30s and 72 °C for 1 min with a final extension of 72 °C for 7 min. The qRT-PCR reactions, had total volume of 10 µl with 10 mM of each gene specific primers, diluted ROX, 1 µl cDNA (1/5 dilution), Brilliant-III ultra-fast SYBR Green qPCR master mix (Agilent Technologies, Santa Clara, CA, USA) and RNase-free water. The experiment was performed on AriaMX Real-time PCR system (Agilent Technologies, Santa Clara, CA, USA). To check the specificity of PCR, amplification melt-curve analysis was carried out at the end of the PCR. Three biological and two technical replicates were used for each experiment and analysis was done by Livak method [60]. Results were expressed as fold change with reference to the control samples.

### 4.8. Statistical Analysis

The data in all figures were expressed as mean ± standard error (SE). Normality and homogeneity of variances were tested through Kolmogorov–Smirnov and Levene testing, respectively. One-way analysis of variance (ANOVA) was conducted to understand the differences among the genotypes under both well-irrigated and drought treatments. Tukey’s test was conducted for mean comparisons. All these were carried out using SPSS package v.19 (IBM SPSS Statistics Version 19.0. Armonk, NY).

### 4.9. Sequence Analysis

Whole genome sequence resource of these three mutants and the WT are readily available with us [54]. Structural variations between the WT and the three mutants were identified for the 15 candidate genes selected for expression analysis in this study. Structural variations in 65 more genes, implicated in literature for SG phenotype, were also analyzed. High quality reads from the mutants were mapped directly on to the N22 genic sequence assembly using bwa v0.7.12 [61]. The sequences of the candidate genes from the mutants and WT were extracted using bedtools getfasta [62]. SNPs were called and amino acid changes were identified using in-house python script.

## 5. Conclusions

We have reported here a promising and novel functional stay-green mutant which has better harvest index under both irrigated and drought conditions. This mutant can be a useful source for crop improvement when combined with high yielding and agronomically superior backgrounds. Our current efforts in mapping this mutant would be useful in understanding the molecular mechanism as well as its utilization. 

## Figures and Tables

**Table 1:** Non-synonymous mutations observed in SGM-1 and SGM-2 in the eight of the 15 candidate genes. SGM-3 had no sequence variation in any of the 15 candidate genes. For more details refer to Table S4.

S. No.	Gene Name	No. of Non-Synonymous (NS) Mutations		Details of NS Changes (Amino Acid Positions and Changes from WT to Mutant)
		SGM-1	SGM-2	
1	*NYC3*	2	2	C→S (15); C→S (465)
2	*Fd-GOGAT*	5	5	I→V (765); I→L (1293); A→V (1302); Q→P (1363); V→I (1417)
3	*NYC1*	1	-	P→R (54)
4	*ATG5*	1	-	L→P (122)
5	*SGR*	-	4	R→C (33); D→N (35); T→A (223); V→A (228)
6	*RCCR1*	-	4	S→C (146); V→L (223); F→V (227); Y→* (Stop codon) (330)
7	*CHL*	-	5	A→G (86); Y→N (148); T→A (217; P→L (237); V→A (356)
8	*GR3*	-	2	N→D (132); N→D (311)

## Supplementary Materials

The following are available online at https://www.mdpi.com/2223-7747/8/10/375/s1. Table S1: Details of the molecular characterization of the SG mutants for their monomorphism with their wild type, Nagina 22 (N22); Table S2: Delta cT values from the time-course relative gene expression analysis of 15 genes involved in chlorophyll catabolism, senescence and nitrogen assimilation in three stay-green mutants (SGM-1 to SGM-3) and the wild type, Nagina 22 (N22) under dark incubation; Table S3: Fold change in enzyme activities under drought stress with respect to well-watered (control) condition; Table S4: Details of the non-synonymous mutations observed out of the additional 65 genes reported in literature to be involved in leaf senescence; Table S5: The protein stability analysis performed for the non-synonymous mutations in the 3Mb QTL region of chromosome 9 didn’t converge to significant outcome; Table S6: Details of the 15 genes involved in chlorophyll catabolism, senescence and nitrogen assimilation and chosen for relative gene expression studies in the mutants and N22.

## Abbreviations

CGs: Candidate genes; DIS: Dark induced senescence; NPT: Number of productive tillers; N22: Nagina22; OS: Oxidative Stress; QTL: quantitative trait loci; SGM: Stay-green mutant; WT: wild-type.
